# Could Exosomes be a Promising Therapy to Accelerate Wound Healing and Skin Regeneration in Ischemic Nasal Flaps?

**DOI:** 10.1093/asjof/ojaf060

**Published:** 2025-06-11

**Authors:** Jenny Carvajal, Melissa Carvajal

## Abstract

Severe nasal soft tissue avulsions often involve skin flaps with compromised vascularity. Ischemic changes ranging from epidermolysis to full-thickness necrosis can occur. Angiogenesis is a critical step in wound healing and tissue repair as well as a key factor in skin flap survival. In previous studies, the authors show that angiogenic capacity declines with age, slowing wound healing. Regenerative medicine is rapidly evolving. Stem cell–derived exosomes are gaining popularity in this field, and potential benefits to stimulate angiogenesis and tissue regeneration, accelerating healing, have been demonstrated in preclinical studies. Therapy could hypothetically be targeting exosomes to deliver proangiogenic factors directly to the ischemic skin flap in the early stage of healing, when inflammation and angiogenesis occur. We present the case of a 75-year-old man with a nasal soft tissue partial degloving injury of the dorsum and tip; the avulsed flap was rolled on itself at the nasal tip for 3 h, showing marked signs of venous congestion. The flap was unrolled and repositioned, but venous congestion persisted, evolving into partial tissue necrosis, which was treated with Rosa Damascena stem cell–derived exosomes (RSCEs) on postinjury Days 6, 11, and 16. Wound healing was achieved with complete skin regeneration at 30 days postinjury. In this case, RSCEs were safely utilized to treat a posttraumatic ischemic nasal flap, suggesting their potential as a therapeutic option to support healing and improve outcomes. Randomized, controlled clinical trials are required to validate this preliminary finding.

**Level of Evidence:** 4 (Therapeutic)

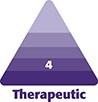

Severe nasal soft tissue avulsions can cause conspicuous deformities or scars, affecting patients’ self-esteem. These injuries often involve skin flaps with compromised vascularity, where disruption of subdermal capillaries and altered microcirculation can lead to ischemia because of arterial insufficiency and venous congestion.^1^

Early evaluation of flap viability and preserving any soft tissue connection is essential. Narrow pedicles increase the probability of venous congestion and possible ischemic complications ranging from epidermolysis to full-thickness necrosis.^2^ Patients should be warned about the possibility of partial or superficial tissue loss.^3^ If necrosis seems inevitable, waiting for demarcation is necessary, because part of the flap may remain viable. Subsequent management may involve secondary healing or reconstructive surgery, depending on defect size, location, and the patient's expectations.^4^

Regenerative medicine is rapidly evolving; stem cell therapy, platelet-rich plasma (PRP), and exosomes are becoming key tools in skin rejuvenation and repair. Exosomes are nonimmunogenic and free of tumorigenic potential compared with stem cell therapy.^5^ Although no direct comparisons between PRP and exosomes exist, the researchers suggest PRP efficacy declines with age because of reduced growth factors and platelet concentration found in elderly patients.^6^

Exosomes are 30 to 200 nm extracellular vesicles derived from the rough endoplasmic reticulum and secreted by cells, carrying bioactive cargo from their parent cells, mediating intercellular communication, and modulating immune responses. Functioning as paracrine effectors of stem cells, exosomes influence target cells at transcriptional and translational levels, delivering messenger RNA (mRNA), microRNA (miRNA), cytokines, and other signaling molecules through mechanisms that remain poorly understood.^7^

Commercial exosome preparations are derived from mesenchymal stem cells obtained from placenta, umbilical cord, adipose tissue, bone marrow, and even plant tissue. Although clinical trials are still lacking, the authors of preclinical studies—primarily using adipose-derived stem cell exosomes (ADSC-Exos)—have shown promising effects promoting angiogenesis, enhancing tissue regeneration, and accelerating healing in necrotic wounds.^8^

Unfortunately, ADSC-Exos are not yet available in many countries; however, plant-derived exosomes have revolutionized the skincare industry and are widely available in the market; the most well-known are Rosa Damascena stem cell–derived exosomes (RSCEs). In 2023, Won et al demonstrated that RSCEs enhance skin cell function by promoting fibroblasts’ proliferation, stimulating collagen synthesis, and reducing inflammation.^9^

The authors of this report aim to explore the use of exosomes as a potential therapy to stimulate skin and soft tissue regeneration after partial ischemic loss of nasal skin flaps, analyzing their role in accelerating healing and improving aesthetic outcomes.

## CASE PRESENTATION

**Figure 1.**
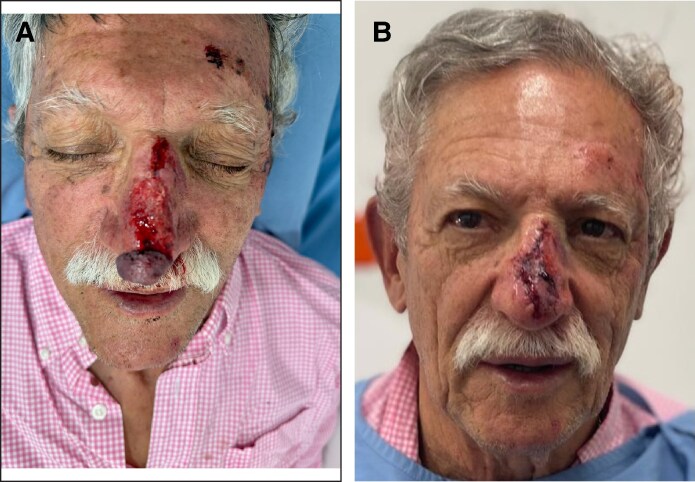
(A) A 75-year-old man, who, 3 h before, had blunt force trauma to nasal soft tissues with partial degloving injury involving the distal two-thirds of the dorsum and part of the nasal tip with exposed cartilage (5 cm × 2 cm). The avulsed flap was rolled on itself with severe venous congestion. (B) The flap and pedicle (1.5 cm wide) were unrolled, but venous congestion persisted. The flap was returned to its original position using 5/0 polypropylene suture.

A 75-year-old man with a medical history of Type 2 diabetes, hypertension, heart failure, transient ischemic attacks, and dyslipidemia arrived at the emergency room 3 h after sustaining blunt force trauma to the soft tissues of his nose following a fall on a rocky trail. Examination revealed a partial degloving injury involving the distal two-thirds of the dorsum and part of the nasal tip. The wound measured ∼5 cm × 2 cm with exposed cartilage. The avulsed flap was rolled on itself at the nasal tip with severe venous congestion and crushed at the distal end (Figure 1A).

Preoperative prophylaxis with Cefazolin 2 g IV was performed. The wound was irrigated, and small foreign bodies were removed; the flap and its 1.5 cm-wide pedicle were unrolled; however, venous congestion remained. The crushed tissue was excised, and the flap was repositioned using 5/0 polypropylene suture (Figure 1B). The patient was discharged with a 5 day course of Cephalexin. Because of persistent venous congestion, 7 sessions of hyperbaric oxygen therapy (HBOT) were recommended; however, only 2 sessions were completed on Days 2 and 3 postinjury.

**Figure 2.**
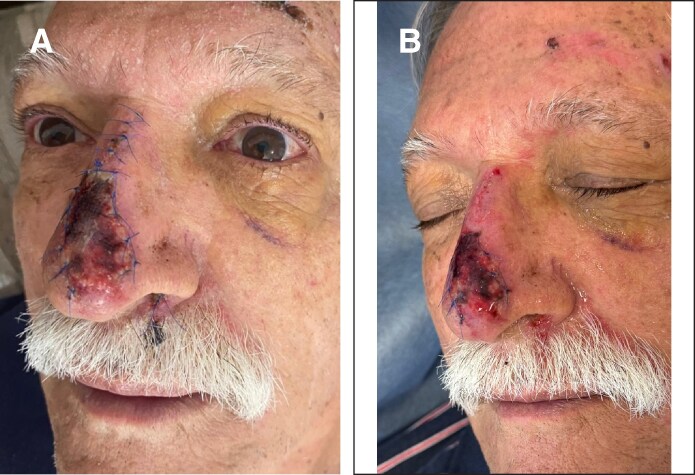
(A) A 75-year-old man, 6 days postnasal degloving injury, skin loss and irregular subcutaneous tissue necrosis within the flap were observed. (B) Six days postnasal degloving injury, debridement of skin and subcutaneous tissue necrosis within the flap was performed, 1 cc of Rosa Damascena stem cell–derived exosomes (ASCEplus Dermal Signal Kit SRLV, ExoCoBio, Korea) was applied to the wound surface. A 3M Adaptic nonadhering dressing was placed for 5 days.

On postoperative Day 6, follow-up revealed skin loss and patchy subcutaneous tissue necrosis within the flap (Figure 2A). After superficial debridement, 1 cc of RSCEs (ASCEplus Dermal Signal Kit SRLV, ExoCoBio, Seoul, Korea) was applied to the wound surface. A 3M Adaptic (Systagenix, Quincy, MA) nonadherent dressing was placed and left for 5 days (Figure 2B).

**Figure 3.**
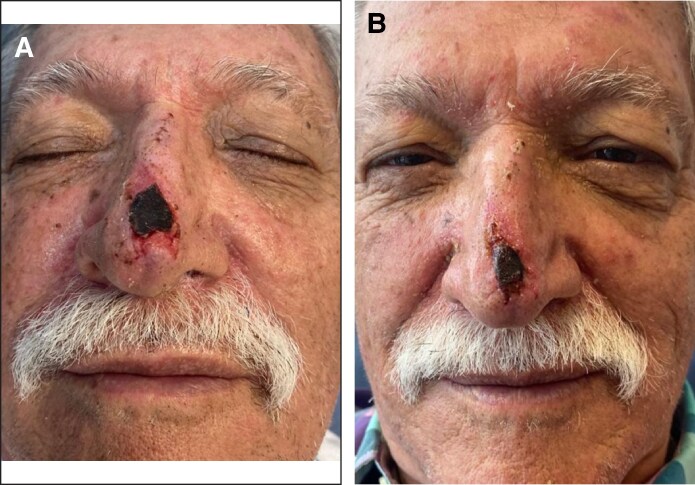
(A) 75-year-old man, 11 days postnasal degloving injury, a peripherally detached eschar was observed. The nonviable tissue was excised, showing early regeneration of the peripheral skin and deeper soft tissue layers. A central portion of the eschar remained adherent. A second 1 cc dose of RSCEs was applied to the wound and covered for 5 additional days. (B) Sixteen days postnasal degloving injury, substantial regeneration of skin and soft tissues was observed. The wound edges become nearly imperceptible, and the central eschar decreased in size. A third 1 cc dose of RSCEs was applied.

On postoperative Day 11, sutures were removed, showing a peripherally detached eschar. Nonviable tissue was excised, exposing early regeneration of the peripheral skin and deeper soft tissue layers. A central portion of the eschar remained adhered. A second 1 cc dose of RSCEs was applied to the wound, which remained covered for 5 additional days (Figure 3A).

By postoperative Day 16, substantial skin and soft tissue regeneration was observed. The wound edges became almost imperceptible, and the central eschar decreased in size. A third 1 cc dose of RSCEs was administered (Figure 3B).

**Figure 4.**
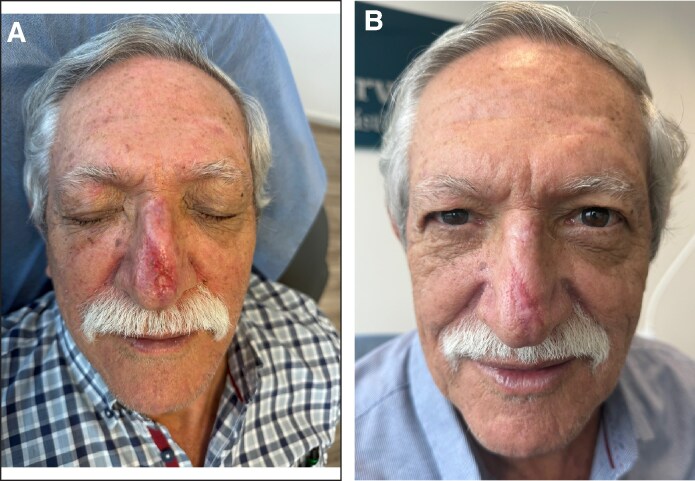
(A) A 75-year-old man, 20 days postnasal degloving injury (14 days after the initial Rosa Damascena stem cell–derived exosome application), re-epithelialization on the surface flap and a trap-door scar deformity on the left side of the nasal tip were observed. (B) Thirty days postnasal degloving injury (24 days after the initial RSCEs application), the wound was completely healed, presenting an immature scar measuring 1.5 cm × 0.4 cm in the area where previous deep necrosis was observed.

**Figure 5.**
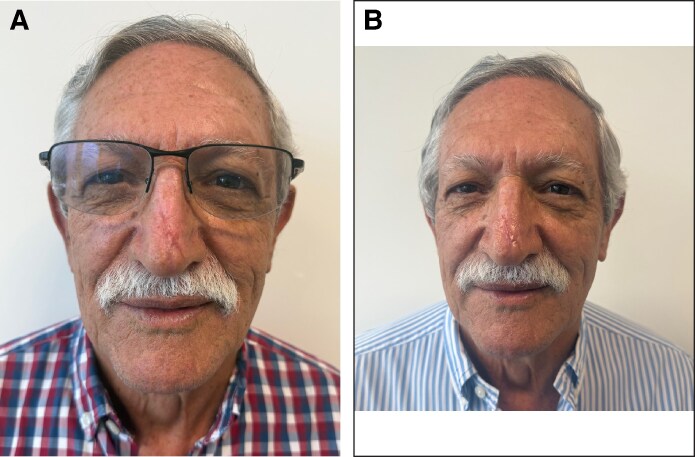
(A) A 75-year-old man, 60 days postnasal degloving injury, the scar continued shrinking progressively. (B) Ninety days postnasal degloving injury, it appeared narrow and barely notable.

**Figure 6.**
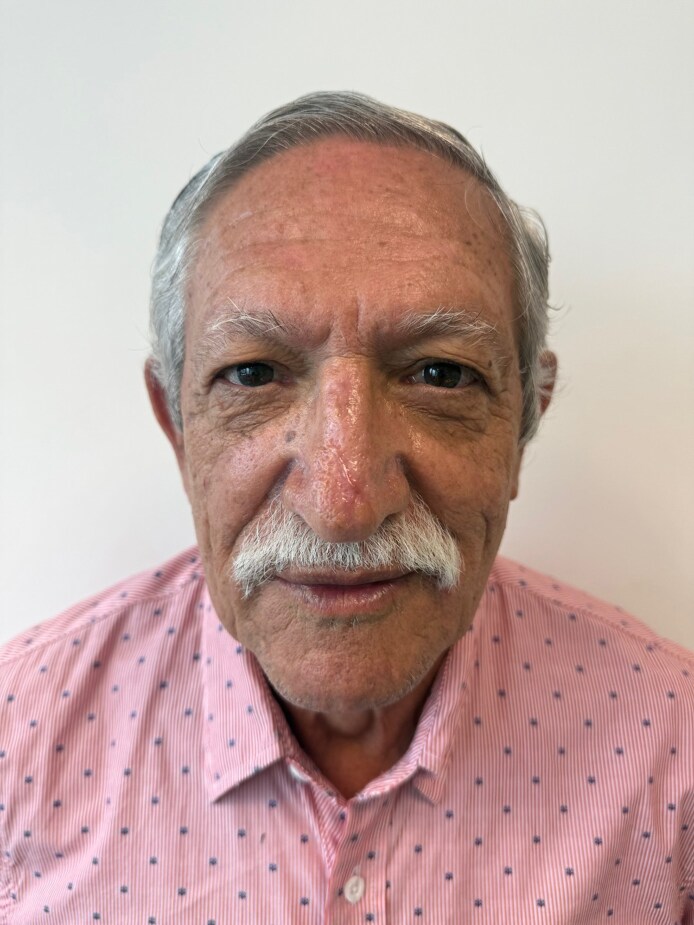
A 75-year-old man, 5 months postnasal degloving injury, reduced scar, and a slight trap-door scar deformity on the left side of the nasal tip was still observed. However, the patient expressed high satisfaction with the aesthetic result.

On postoperative Day 20 (14 days after the initial RSCE application), re-epithelialization was evident, with a small trap-door scar on the left side of the nasal tip (Figure 4A). By Day 30, the wound had completely healed, presenting a 1.5 cm × 0.4 cm immature scar in the central area where deep necrosis had been previously (Figure 4B). The scar continued to contract and progressively improved over time (Figure 5A). At 3 months, it appeared narrow and barely notable (Figure 5B). By 5 months, the patient expressed high satisfaction with the aesthetic result; however, a reduced scar and a slight trap-door deformity on the left side of the nasal tip were still observed (Figure 6).

## DISCUSSION

Random-pattern skin flaps receive their blood supply from the subdermal vascular network. This low-pressure system can be compromised by excessive twisting or extension of the flap.^1^ In this patient, a long avulsed nasal skin flap was rolled up, resulting in mechanically limited venous outflow for 3 h, making the prognosis questionable. Venous congestion quickly compromises flap viability, leading to irreversible failure within 3 h.^4,10^

Although reconstruction of aesthetic nasal subunits remains controversial, recent literature favors maximal conservation of native tissue.^11^ Bevans advocates preserving partially avulsed tissue because of the nose's excellent collateral circulation, highlighting the risk of venous congestion and partial or complete flap necrosis.^3,12^

Little evidence supports the use of HBOT for flap venous congestion. The authors of preclinical studies show conflicting results, and 1 randomized clinical trial found no clear benefit.^13^ Two case reports describe partial flap salvage after 7 to 12 days of HBOT following replantation of nonvascularized nasal tissue.^3^ Thus, in this case, 2 sessions were likely insufficient to impact the outcome.

It is well-known that angiogenesis is a critical step in wound healing and tissue repair, as well as a key factor in skin flap survival.^14,15^ In ischemic flaps, the problem is insufficient neovascularization and ischemia-reperfusion (I/R) injury, which are mainly responsible for poor healing results.^16^

In previous studies, the authors show that angiogenic capacity declines with age, slowing wound healing.^17^ Aging impairs tissue repair through altered inflammation, reduced collagen synthesis, delayed angiogenesis, and slower epithelialization.^18^ Reduced levels of fibroblast growth factor, vascular endothelial growth factor (VEGF), and transforming growth factor have been implicated as partially responsible for delayed angiogenesis. Indeed, replenishing these growth factors can reverse slow neovascularization.^19^

Aging and comorbidities may have impacted ischemic flap survival in this case. Dermal microcirculation decreases in elderly patients with hypertension and dyslipidemia.^20^ There is also an association between impaired angiogenesis and pathologic wound repair in diabetic patients.^21^

Growing preclinical evidence indicates that exosomal miRNAs modulate pathways in the inflammatory, proliferative, angiogenic, and remodeling phases of wound healing.^22^ The authors of recent studies highlight the role of many differentially expressed miRNAs in ADSC-Exos to enhance vascularization of skin flaps with insufficient angiogenesis, improving survival.^23^ Pu et al demonstrated that interleukin (IL)-6-rich human ADSC-Exos promoted flap angiogenesis and repair after I/R injury in mice.^24^ Many researchers agree that IL-6 plays a key role in wound healing with oxidative damage.^25^

RSCEs have been shown to contain biomolecules, such as RNAs, proteins, and lipids, which regulate skin cell functions similar to human exosomes.^26^ In an in vitro study, RSCEs were shown to close wounds, likely by stimulating fibroblast proliferation and migration, as well as the intracellular delivery of biomolecules including RNA, although their mechanism remains unclear.^(27)^ Bioinformatics analysis of RNA content in RSCEs suggests that miR-23 may promote angiogenesis.^9^

Kim et al evaluated the efficacy of rose placenta extract obtained from Rosa Damascena for full-thickness wound healing in mice. Greater expression of epidermal growth factor and VEGF was observed with early stimulation of angiogenesis, leading to accelerated healing with statistically significant results.^28^

Given preclinical research, Kim's study, the critical condition of the nasal flap, the patient's age and comorbidities, and the availability of RSCEs, we opted for off-label use of RSCEs as the last resort to salvage the flap.^29^ Although ADSC-Exos would have been ideal because of their potential efficacy in ischemic skin flap repair, they were unavailable. A growing number of publications on plant-derived exosomes point to them as an effective alternative to those from allogeneic and xenogeneic sources, although the number of registered clinical trials using this therapy remains very limited.^29^

Clinical studies to establish optimal exosome therapy for skin injury and wound healing are ongoing.^30^ In studies with animal models, exosomes have been utilized to treat skin injury with doses ranging from 1 to 200 μg per treatment, applied topically to the surface or incorporated into a wound dressing to promote healing.^30^ Several general guidelines have been suggested based on existing preclinical studies, which recommend applying exosomes at intervals ranging from daily to weekly.^31^ Exosome therapy could vary depending on the desired therapeutic goal. In theory, a single dose at the time of injury or at the initial phase of wound healing could effectively promote tissue regeneration, whereas repeated administration—at fixed intervals or as needed—could support progressive tissue repair.^30^

VEGF levels peak between Days 3 and 7 post-full-thickness injury, coinciding with granulation tissue formation.^32^ Therapy could hypothetically be targeting exosomes to deliver proangiogenic factors directly to the ischemic skin flap during this window in the early stage of healing, when inflammation and angiogenesis occur.^30^ In this case, RSCEs were applied on the sixth day postoperative to improve angiogenesis and enhance survival of the ischemic nasal flap.

Exosome applications were also performed on postoperative Days 11 and 16 to improve healing and aesthetic outcome. Exosomes may promote cell growth and tissue regeneration during the proliferative phase and help promote the organization of new tissue in the remodeling phase. They also contribute to improve skin texture and tone, as well as promote collagen and elastin production in aged skin.^30^ The question arises as to whether similar results could have been achieved without exosome intervention in this patient.

Exosome therapy is an emerging field. To date, the use of commercial preparations in skin injuries remains off-label. Although promising, exosomes may not fully replicate the regenerative capacity of stem cells, particularly in severe tissue damage. Potential side effects and long-term outcomes require further investigation. Cost may also limit accessibility, especially if multiple doses are required. Although exosomes show potential in enhancing wound healing and regeneration in ischemic nasal flaps, robust evidence from randomized controlled clinical trials is still needed to validate these preliminary findings.

## CONCLUSIONS

Ischemic skin flap failure can be a devastating complication in both cosmetic and reconstructive nasal surgery. In multiple preclinical studies, the authors suggest that exosomes may enhance angiogenesis, reduce ischemic damage, and promote wound healing and tissue regeneration. In this case, RSCEs were safely utilized to treat a posttraumatic ischemic nasal flap, suggesting their potential as a therapeutic option to support healing and improve outcomes.
